# Distinct Skin Microbiota Imbalance and Responses to Clinical Treatment in Children With Atopic Dermatitis

**DOI:** 10.3389/fcimb.2020.00336

**Published:** 2020-07-03

**Authors:** Ying Liu, Shan Wang, Wenkui Dai, Yuan Liang, Chunping Shen, Yunzhu Li, Lei Jiao, Yawei Bian, Zhan Gao, Yinhu Li, Dongfang Li, Shuaicheng Li, Martin J. Blaser, Yi-Wei Tang, Lin Ma

**Affiliations:** ^1^Department of Dermatology, Beijing Children's Hospital, Capital Medical University, National Center for Children's Health, Beijing, China; ^2^Department of Computer Science, City University of Hong Kong, Hong Kong, China; ^3^Center for Advanced Biotechnology and Medicine, Rutgers University, New Brunswick, NJ, United States; ^4^Department of Microbial Research, WeHealthGene Institute, Shenzhen, China; ^5^Department of Laboratory Medicine, Memorial Sloan Kettering Cancer Center, New York, NY, United States; ^6^Department of Pathology and Laboratory Medicine, Weill Medical College of Cornell University, New York, NY, United States

**Keywords:** atopic dermatitis, skin microbiota imbalance, clinical treatment, children, China

## Abstract

**Background:** Atopic dermatitis (AD) is a common cutaneous disease, associated with imbalances in the skin microbiota.

**Objective:** To explore the characteristics of the cutaneous microbiota and its dynamic changes during clinical treatment.

**Methods:** Cutaneous swab samples were collected from 51 AD patients before treatment, and 40 AD patients remained after a 2-week treatment with mometasone and mupirocin.

**Results:** AD patients exhibited significant enrichments of *Prevotella* and *Desulfovibrio* as well as obvious reductions of *Corynebacterium, Streptococcus* and *Parabacteroides*. Based on the proportion of *Staphylococcus aureus*, the AD patients were further classified into *S. aureus-*predominant group (AD.S) and *S. aureus-*non-dominant (AD.ND) group. The AD.S group exhibited lower skin microbial diversity and higher atopic dermatitis (SCORAD) index. In the AD.S group, the cutaneous microbial diversity significantly increased from 2.9 ± 0.8 to 3.7 ± 1.0, while the relative abundance of *S. aureus* decreased from 42.5 ± 20.7 to 10.3 ± 28.4 after treatment. In contrast, no significant skin microbiota changes were detected in the AD.ND group.

**Conclusions:** AD patients with predominant *S. aureus* had higher disease severity and lower microbiota diversity compared to patients in the AD.ND group. Mometasone and mupirocin therapy had significant effects on skin microbiota in AD.S patients, but had a paradoxical response in the AD.ND patients.

## Introduction

Atopic dermatitis (AD) is a chronic, relapsing and pruritic inflammatory skin disease, with a prevalence of 10–20% in children worldwide (Weidinger and Novak, 2016). Among affected patients, 60% develop AD before 1 year old (Illi et al., 2004), and flare-ups occur in 85% within the first 5 years of life (Sampson, 1999). Although there is a clear genetic predisposition to disease (Wollenberg et al., 2018), AD flare-ups also have been associated with environmental factors, including allergen exposure, skin barrier defects, cutaneous and intestinal microbiota characteristics, as well as seasonal rhythms (Hulshof et al., 2017). Importantly, the incidence of AD has rised progressively wherever it has been studied longitudinally (Abuabara et al., 2018), consistent with changing environmental triggers and/or causation (Abuabara et al., 2018). One possibility is that immune imbalances leading to AD are mediated by a changing human microbiota, which is especially important during the early years of life (Blaser, 2017). Differences in the cutaneous microbiota between peoples with traditional lifestyles and those from industrialized countries support this hypothesis (Blaser et al., 2013; Clemente et al., 2015).

Current therapeutic strategies for AD include moisturizer, corticosteroid, antibiotics and/or calcineurin inhibitor (Ma et al., 2017). Despite substantial efficacy (Ma et al., 2017), long-term administration of antibiotics and/or antihistamines might affect the development of children (Wollenberg et al., 2018) and raise the likelihood of antibiotic resistant pathogens (Werfel et al., 2009). The use of probiotics has shown efficacy in some AD treatment trials due to their effects on host immunity (Zhao et al., 2018), but clinical utility has been uneven (van der Aa et al., 2010; Zhao et al., 2018).

In recent years, there was an explosion of knowledge about the cutaneous microbiota in healthy subjects (Gao et al., 2007; Costello et al., 2009; Findley et al., 2013). Because of the roles of the microbiota in educating the immune system and inducing or controlling inflammatory responses (Chng et al., 2016; Eyerich et al., 2018), there was especial interest in the cutaneous microbiota in AD (Kong et al., 2012; Gonzalez et al., 2016; Byrd et al., 2018). Prior studies have shown cutaneous microbiota alterations in AD patients, including decreased microbial diversity, enriched *Staphylococcus* and depleted *Dermacoccus* (Kong et al., 2012; Chng et al., 2016). Altered bacterial compositions (termed dysbiosis) might elicit intense inflammatory and immune responses in AD patients (Chng et al., 2016), and aggravate skin injuries through metabolic alterations and pH elevation (Byrd et al., 2018). Greater knowledge of the dynamic changes in microbiota in the context of clinical treatment is needed.

In the current study, we recruited 51 AD patients and 31 healthy children (HC) to characterize the cutaneous microbiota. In addition to exploring the differences in skin microbiota between these two groups, we explored the dynamic changes due to therapy in relation to both microbial components and clinical outcomes.

## Materials and Methods

### Ethics Statement

This study was approved by the Ethics Committee of Beijing Children's Hospital, Capital Medical University under registration number IEC-C-008-A08-V.05.1. All procedures were conducted according to the guidelines stipulated by the Ethics Committee of Beijing Children's Hospital, and all clinical investigations were conducted according to the principles expressed in the Declaration of Helsinki. The parents of all children provided written informed consent, volunteering for investigation of their children for scientific research.

### Participant Recruitment

AD patients were recruited from the Department of Dermatology, Beijing Children's Hospital of Capital Medical University with the following inclusion criteria for study subjects: (I) between 2 and 12 years old; (II) clear clinical manifestations of AD according to Williams criteria (Williams, 2005); and (III) moderate and severe AD patients were selected when their scoring atopic dermatitis index (SCORAD) exceeded 25 (Taieb and Stalde, 1993). The SCORAD was adopted to evaluate the severity of AD with both objective and subjective components, such as the extent of skin lesion, the intensity of skin lesion, the degree of itching and sleep disorders (Supplementary File 1). HC were selected from those who passed physical examinations of Beijing Children's Hospital of Capital Medical University with the following standards: (I) between 2 and12 years old; and (II) no allergic history (e.g., including food allergy, AD, asthma, allergic rhinitis and allergic conjunctivitis). Both AD patients and HC were excluded from the study if: (I) they had been exposed to bleach bath, corticosteroid, antibiotic, probiotic, or proton pump inhibitor within 4 weeks before skin sample collection; (II) known hereditary disease (e.g., thalassemia, hereditary deafness, phenylketonuria); (III) known metabolic or autoimmune disease (e.g., obesity, diabetes, rheumatoid arthritis). In total, 51 AD patients and 31 HC were enrolled between February 2016 and March 2017 (Supplementary File 1).

### Cutaneous Samples Collection

The skin samples were collected from the lesions at anticubital fossa of participants by using Copan flock swabs (4N6FLOQSwabs, Copan Diagnostics Inc., Murrieta CA, U.S.A.) which were moistened with DNA-free water (Nuclease-Free Water, Qiagen-China, Shanghai, China). The diameter of the sampling area was <2 cm^2^. Initially, the affected skin was stroked 50 times in the direction of left and right using a cotton swab. Then, the same affected skin was stroked another 50 times in the direction of up and down using the same cotton swab. As described in previous clinical studies (Lyons et al., 2015; Wollenberg et al., 2018), the AD patients were treated with the mixture of topical mometasone (Eloson, Bayer-China, Shanghai, China) and mupirocin (Bactroban, Glaxo Smith Kline-China, Shanghai, China). These two drugs were mixed at a1:1 ratio and applied twice daily directly to the lesions. After 2-week's treatment, 40 AD patients remained for the collection of cutaneous samples.

### DNA Extraction, Library Construction, and Sequencing

Microbial DNA was extracted from cutaneous samples using the E.Z.N.A® Soil DNA Kit (Omega Bio-tek, Norcross GA, U.S.A.) according to the manufacturer's protocols. Using PCR kit (AP221-02, TransGen Biotech, Beijing, China), the V3-V4 region of the bacterial 16S rRNA gene was amplified by primers 338F and 806R, and the quality of the PCR products was checked by Qubit (Thermo Fisher Scientific-China, Shanghai, China). PCR products were prepared for library construction (TruSeq DNA PCR-Free kit, Illumina, San Diego CA, U.S.A.), and sequenced as 300 (nt) reads using the MiSeq platform (Illumina). Connected tags were uploaded to the NCBI Sequence Read Archive (SRA) Database (Accession number: PRJNA521807).

### Data Filtering and Taxonomical Annotation

To obtain high quality data, the raw reads were removed when they contained more than 10 low-quality (< Q20) bases or 15 bases of adapter sequences (Wang et al., 2016). Based on at least 50 overlapping bases, the filtered reads were connected into tags, and using USEARCH (v7.0.1090; Edgar, 2013), the tags clustered into operational taxonomic units (OTUs) with 97% similarity. The taxonomic positions of the OTUs were identified using the RDP 16S rRNA databases (trainset 16/release 11.5; Cole et al., 2014). The Shannon index and the Bray-Curtis distance between cutaneous microbiota samples were calculated by using package “vegan” in R (version 3.4.1).

### Hierarchical Clustering of the Sequence Data

The construction of a hierarchical clustering tree involved three steps (Costea et al., 2018). First, the Bray-Curtis distances among samples were calculated based on OTU abundances (using package “vegan” in R). Second, samples are clustered by Bray-Curtis distance using the “hclust” package in R (Parameter: method=”average,” and other parameters were set at default), and the output tree file was obtained. Finally, the tree file together with genus profiling file and group information file were visualized by online tool iTol (Letunic and Bork, 2016).

### Statistics

All statistical analysis was performed in R. Wilcoxon rank-sum test (using “wilcox.test”) or Chi-Square test (using “chisq.test”) was applied to detect differences among groups (*P* < 0.05), while Wilcoxon signed-rank test (using “wilcox.test”) was used for the comparison in AD patients before and after treatment (*P* < 0.05). Linear regression analysis was performed to detect the associations between the abundance of *S. aureus* and SCORAD index (using the package “lm” in R). Statistical results from the multiple tests were adjusted with the Benjamini and Hochberg method (*FDR* < 0.05) using the function “p.adjust” in R. The statistical results were plotted using package “ggplot2” in R.

## Results

### Characteristics of the Study Subjects

A total of 51 AD children (AD group) and 31 healthy children (HC group) were enrolled in the study and 40 of the AD patients were available for follow-up investigation and cutaneous microbiota analysis after clinical treatment (AD-treated group). After the mometasone and mupirocin therapy, the SCORAD index in the patients with follow-up decreased substantially (Table 1, *P* < 0.001, Wilcoxon signed-rank test), as expected (Wollenberg et al., 2018). The 16S rRNA sequencing reads from all cutaneous samples accounted for 1,762,507 tags (mean ± SD, depth per sample = 14,447 ± 2,916). The mean (±SD) number of OTUs was 266 ± 188: ranging from 253 to 864 in the HC group (505 ± 205), 77 to 444 in the AD group (185 ± 86) and 87 to 380 in the AD-post-treatment group (184 ± 82). The number of OTUs in the AD group was significantly lower than in the HC group (*P* < 0.001, Wilcoxon rank sum test). After RDP database alignment, 443 genera from 24 phyla were identified in all samples (Supplementary File 2), while the mean (±SD) number of annotated genera were 135.5 ± 35.7, 69.4 ± 20.3 and 70.0 ± 18.0 in the HC, AD and AD-post-treatment groups, respectively. The AD group contained significantly fewer annotated genera than the HC group (*P* < 0.001, Wilcoxon rank sum test). These results confirm the reported dysbiosis (Kong et al., 2012; Chng et al., 2016; Gonzalez et al., 2016) in AD lesional skin.

**Table 1:** Clinical features of the 82 study subjects.

	**HC(*n* = 31)**	**AD (*n* = 51)**	***P*-value**
**SCORAD score**			
Pre-treatment	0	49.2 ± 12.7	<0.001^#^ (Wilcoxon signed-rank test)
After treatment	0	16.9 ± 13.8	

# *Comparing all 51 AD patients before and after treatment*.

### Altered Cutaneous Microbiota in AD Patients

**Figure 1:**
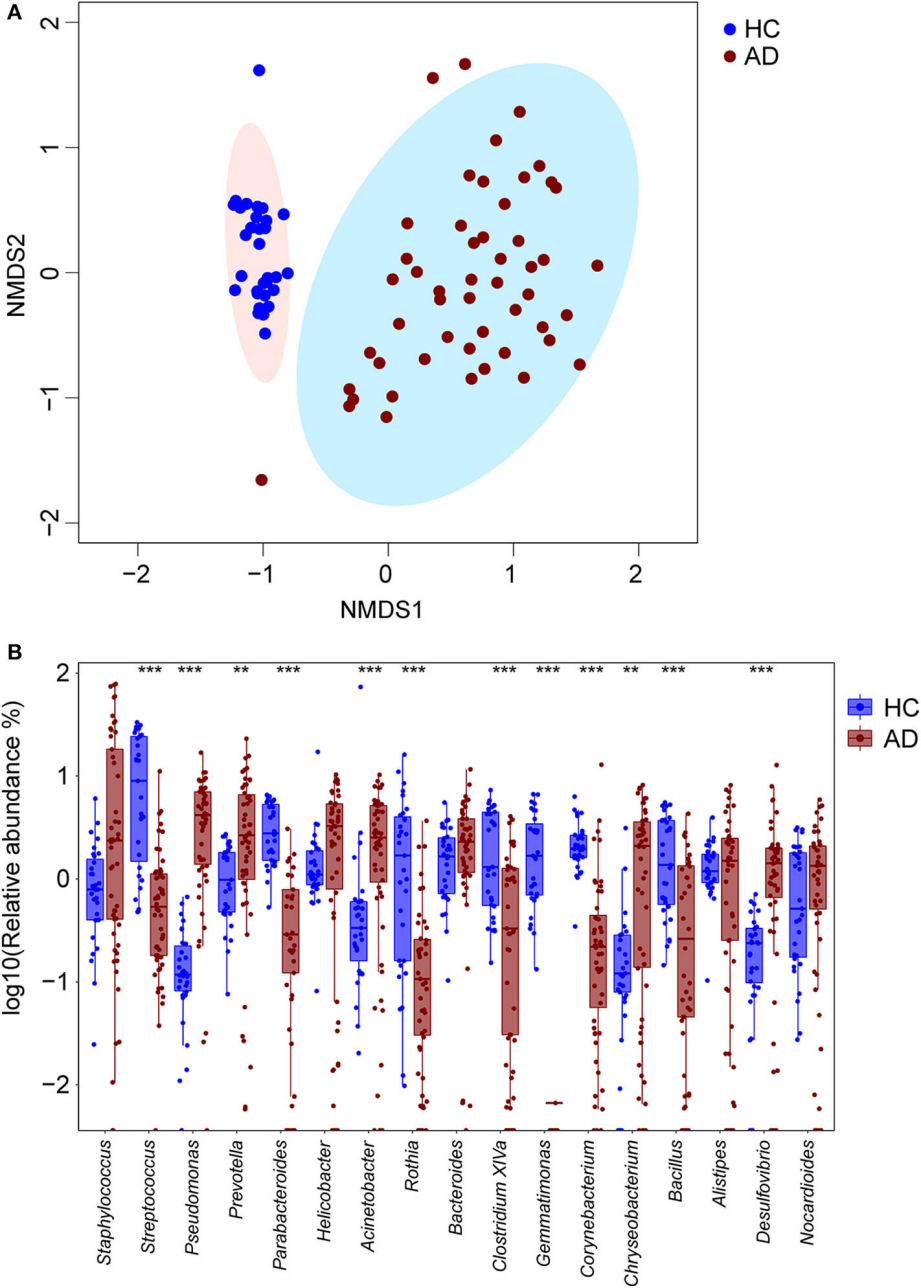
Differing cutaneous microbiota at baseline in HC and AD patients. **(A)** Non-metric multi-dimensional scaling (NMDS) plot of bacteria in cutaneous samples from 31 HC (blue circles) and 51 children with AD (maroon circles). By NMDS analysis, these two groups are distinct. **(B)** The 10 most abundant genera for 31 HC and 51 AD patients were detected, and their log_10_ relative abundances represented with blue and maroon boxes respectively. In each box, the median value suggested the baseline abundances of the genera in the group. Differentially enriched microbial constituents between these two groups were suggested with **P < 0.01 and ***P < 0.001.

With non-metric multidimensional scaling (NMDS), the baseline samples from the AD group separated significantly from those in the HC group (Figure 1A). The most highly represented taxa in the skin microbiota of the HC group were *Streptococcus* (12.7 ± 11.6%), *Parabacteroides* (3.4 ± 2.0%), *Rothia* (2.9 ± 3.7%), *Acinetobacter* (2.9 ± 13.1%) and *Clostridium XIVa* (2.6 ± 2.2%). In contrast, the skin microbiota from the AD group had enriched *Pseudomonas* (4.5 ± 3.6%, *P* < 0.001, *FDR* = 0.000, in comparison with HC, Wilcoxon rank sum test), *Prevotella* (4.5 ± 4.9%, *P* = 0.001, *FDR* = 0.003, Wilcoxon rank sum test), *Acinetobacter* (3.0 ± 2.7%, *P* < 0.001, *FDR* = 0.001,Wilcoxon rank sum test), *Chryseobacterium* (2.2 ± 2.4%, *P* = 0.001, *FDR* = 0.004, Wilcoxon rank sum test) and *Desulfovibrio* (1.7 ± 2.3%, *P* < 0.001, *FDR* = 0.000, Wilcoxon rank sum test). Conversely, the proportions of *Streptococcus* (1.1 ± 1.8%, *P* < 0.001, *FDR* < 0.001, Wilcoxon rank sum test), *Parabacteroides* (0.3 ± 0.6%, *P* < 0.001, *FDR* < 0.001, Wilcoxon rank sum test), *Clostridium XIVa* (0.7 ± 1.0%, *P* < 0.001, *FDR* < 0.001, Wilcoxon rank sum test) and *Corynebacterium* (0.7 ± 1.9%, *P* < 0.001, *FDR* < 0.001, Wilcoxon rank sum test) were reduced in the AD group (Figure 1B). Moreover, the AD patients had lower skin microbial diversity: the average Shannon indexes were 3.87 ± 0.83 and 4.71 ± 0.79 in the AD and HC groups, respectively (*P* < 0.001, *FDR* < 0.001, Wilcoxon rank sum test). Consistent with prior reports (Kong et al., 2012; Chng et al., 2016; Gonzalez et al., 2016), the children with AD in this study had altered taxa.

### AD Patients Can Be Sub-classified Into *S. aureus-*Predominant and *S. aureus-*Non-dominant Groups

**Figure 2:**
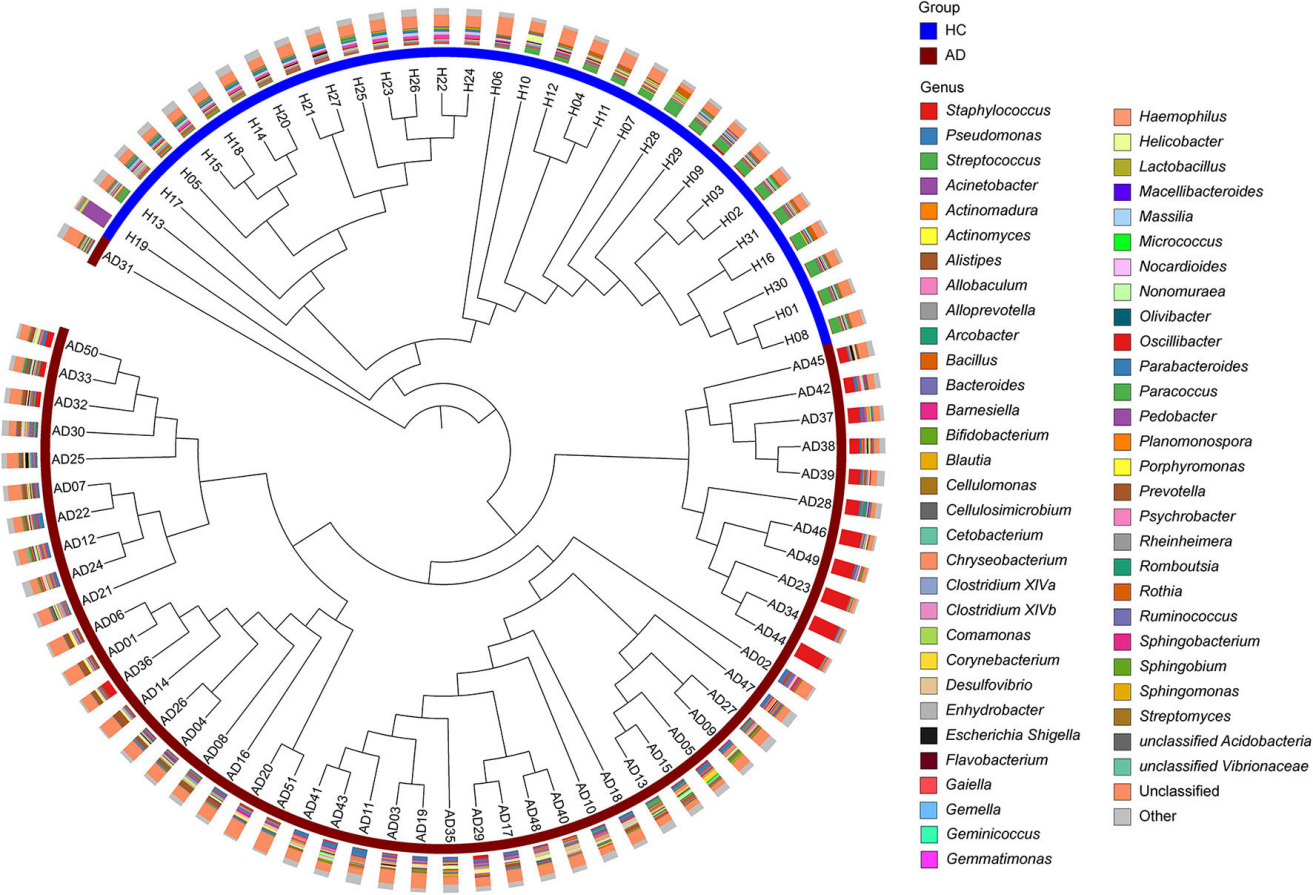
Hierarchical clustering of microbial samples from cutaneous sites. The 82 microbial samples [31 HC (blue) and 51 AD (maroon)] were clustered as determined by the Bray-Curtis distances. In the outer circle, the histograms show the relative abundances of the major microbial constituents in each sample. Three main branches can be identified: (I), samples from HC; (II), *S. aureus-*predominant AD patients (AD.S group), and (III) *S. aureus-*non-dominant AD patients (AD.ND group).

Based on hierarchical analysis, cutaneous microbial samples in the HC group clustered together. Among 51 AD patients, skin microbial populations from 50 patients could be classified into two different sub-clusters (Figure 2). Further analysis indicated the dominance of *S. aureus* (*n* = 11, AD.S group) in one sub-group, whereas skin microbiota in the other (larger) sub-group was not dominated by *S. aureus* (*n* = 39, AD.ND group; Supplementary File 3).

**Figure 3:**
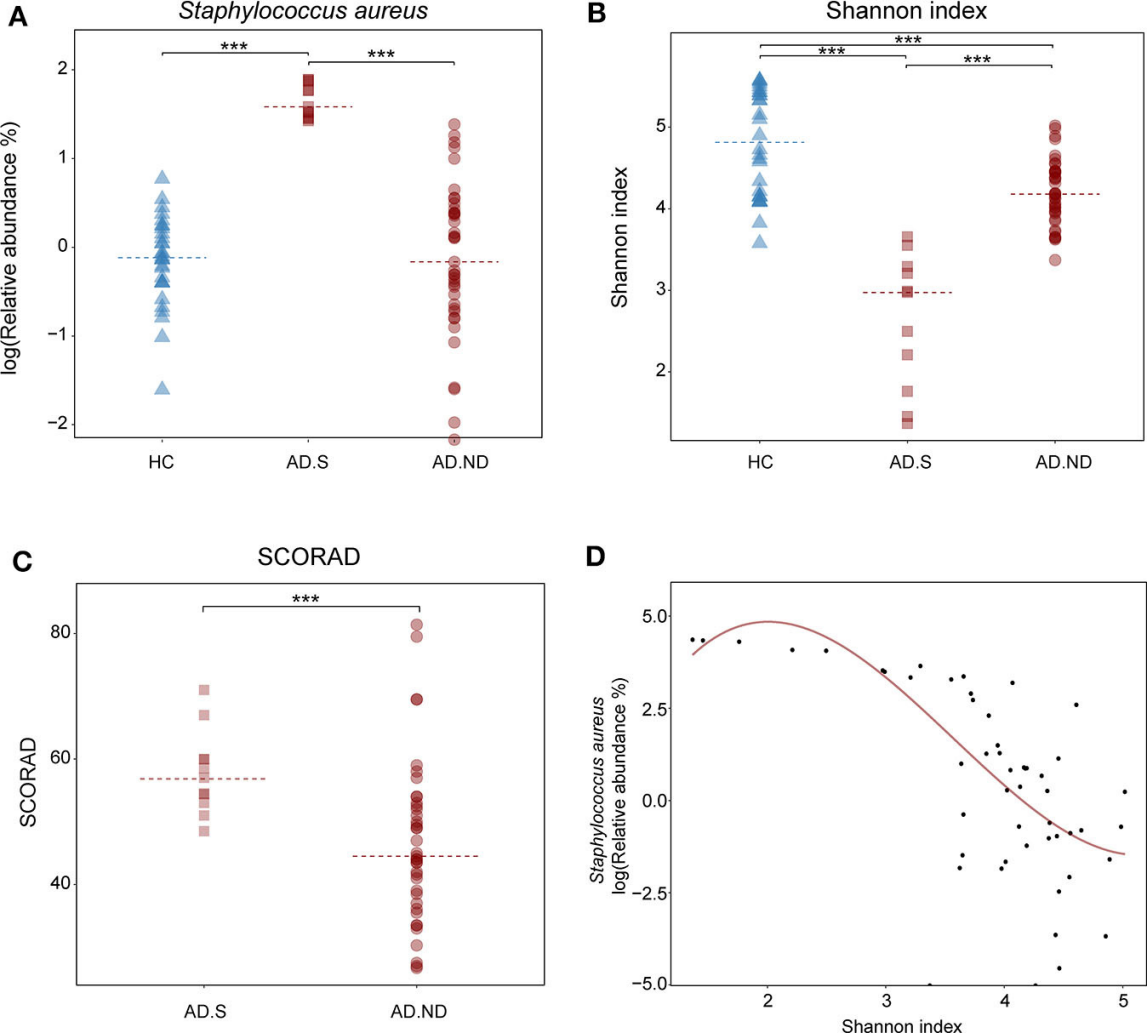
Microbial diversity and disease severity in the HC, AD.S and AD.ND groups. **(A)** Relative abundances of *S. aureus* in the three groups. ***P < 0.001. **(B)** Shannon index, reflecting microbial diversity. ***P < 0.001. **(C)** SCORAD index, reflecting the extent of the cutaneous injury in the AD patients. ***P < 0.001. **(D)** Correlation of cutaneous microbial diversity and relative abundance of *S. aureus*. ***P < 0.001 and *R*^2^ = 0.8. The samples in HC, AD.S and AD.ND groups were represented by blue triangles, maroon squares, and maroon circles, respectively.

In the AD.S and AD.ND groups, the relative abundances of *S. aureus* were 48.7 ± 20.9% and 3.1 ± 5.5% respectively (*P* < 0.001, *FDR* < 0.001, Wilcoxon rank sum test, Figure 3A), whereas there was no significant difference between the HC and AD.ND groups (*P* = 0.77, *FDR* = 0.81, Wilcoxon rank sum test, Figure 3A) in *S. aureus* abundances. The microbial diversity in the AD.S group was significantly lower than in the AD.ND group (*P* < 0.001, *FDR* < 0.001, Wilcoxon rank sum test, Figure 3B), with the proportions of *S. aureus* negatively correlated with the Shannon index (*R*^2^ = 0.8, Figure 3D). The SCORAD index in AD.S (57.7 ± 6.7) was significantly higher than in the AD.ND group (46.7 ± 13.1, *P* = 0.002, *FDR* = 0.002, Wilcoxon rank sum test, Figure 3C). In summary, these findings show that the patients with AD can be divided into two distinct groups, based on *S. aureus* relative abundance.

### *S. aureus-*Predominant and *S. aureus-*Non-dominant Groups Responded Differentially to Clinical Treatment

**Figure 4:**
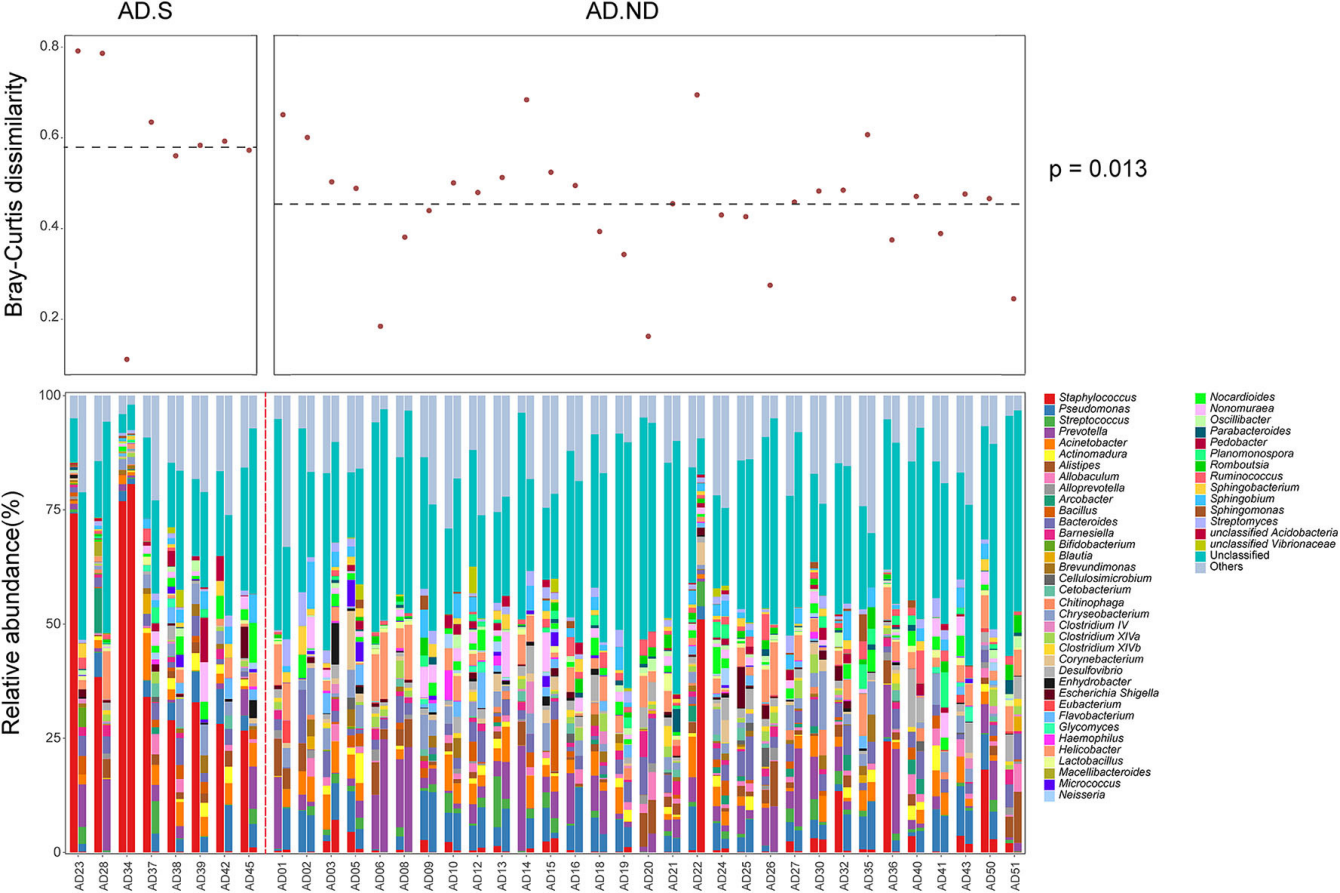
Cutaneous microbiota in AD patients before and after therapy. In the upper panel, the Bray-Curtis distance was calculated for the skin microbiota changes in each AD patient. In the lower panel, the skin microbiota compositions were analyzed in 39 AD patients before and after clinical treatment. The red dotted line was used to separate patients in the AD.S group and AD.ND groups.

**Figure 5:**
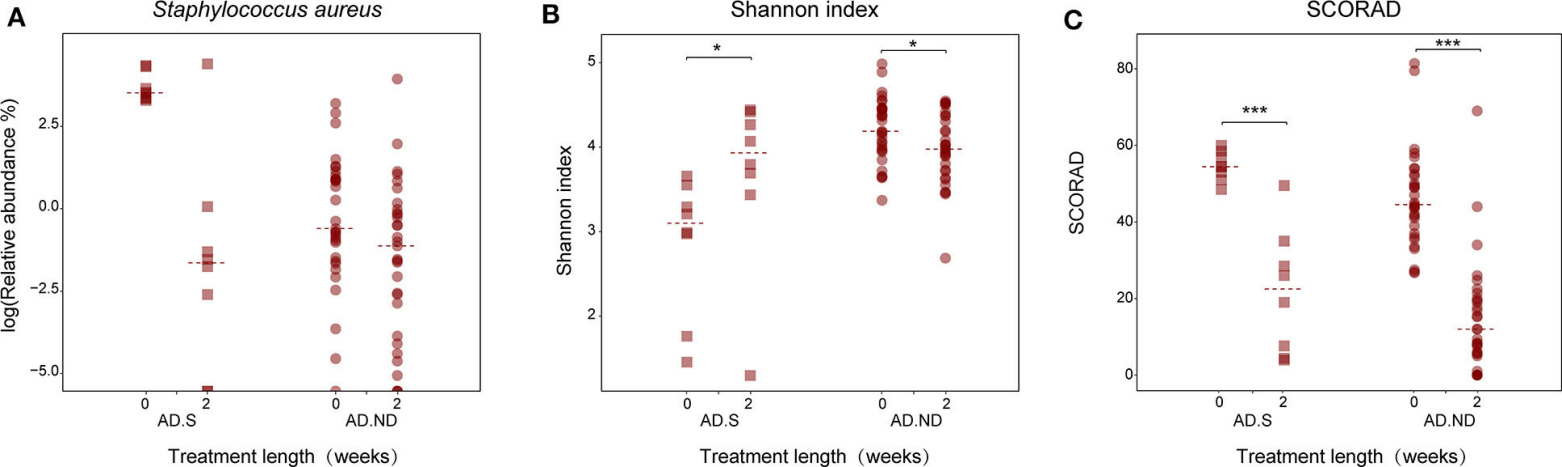
Changes in *S. aureus* abundance, microbial diversity and SCORAD index in the AD.S and AD.ND groups before and after treatment. **(A)** Relative abundances of *S. aureus*. *FDR* > 0.05. **(B)** Shannon index. *P < 0.05. **(C)** SCORAD index. ***P < 0.001. The maroon squares and maroon circles represent samples in AD.S and AD.ND groups, respectively.

After clinical treatment for 2 weeks, alteration of the skin microbiota was investigated in 39 AD patients, from the AD.S group (*n* = 8) or AD.ND group (*n* = 31; Figure 4). The skin microbiota changed in the AD patients after clinical treatment, and more intense alterations were observed in the AD.S group compared with the AD.ND group (*P* = 0.013, Wilcoxon rank sum test, Figure 4). In the AD.S group, a sharp reduction of *S. aureus* was observed in 7 of 8 patients after treatment (Figure 5A), and its mean abundance decreased from 42.5 ± 20.7% to 10.3 ± 28.4% (*P* = 0.014, *FDR* = 0.666, Wilcoxon signed-rank test). However, although the abundance of *S. aureus* decreased in 22 of 31 AD.ND patients, it did not change significantly (from 2.9 ± 5.6% to 2.5 ± 9.1%, *P* = 0.06, *FDR* = 1.0, Wilcoxon signed-rank test) nor did other highly represented skin bacteria (Figure 4). The Shannon index increased from 2.9 ± 0.8 to 3.7 ± 1.0 in the AD.S group (*P* = 0.016, *FDR* = 0.016, Wilcoxon signed-rank test), but decreased from 4.2 ± 0.4 to 4.0 ± 0.4 in the AD.ND group (*P* = 0.032, *FDR* = 0.032, Wilcoxon signed-rank test, Figure 5B). Focusing on the 39 AD patients who were available before and after treatment, the SCORAD value decreased significantly in both the AD.S [54.6 ± 3.8 to 21.7 ± 16.2 (*P* = 0.008, *FDR* = 0.008, Wilcoxon signed-rank test)] and AD.ND groups [46.2 ± 12.8 to 14.6 ± 14.6 (*P* < 0.001, *FDR* < 0.001), Wilcoxon signed-rank test] (Figure 5C). In total, these results indicate the distinctive cutaneous microbiota responses in AD.S and AD.ND groups after clinical treatment.

## Discussion

AD is a chronic relapsing inflammatory skin disease with high prevalence in children (Weidinger and Novak, 2016), triggered by environmental perturbations in genetically predisposed individuals (Hulshof et al., 2017). Since *S. aureus* is highly prevalent on AD skin especially the lesional sites (Brook, 2002; Gonzalez et al., 2016), antibiotics are often used as part of the clinical approach together with anti-inflammatory modalities (Wollenberg et al., 2018). However, since the composition of the cutaneous microbiota has been closely associated with the occurrence of AD (Yamazaki et al., 2017), the relationships of antimicrobial interventions with the particular cutaneous microbiota need to be clarified. In this study, we characterized the cutaneous microbiota of AD patients, and explored the responses to combined treatment with mometasone (anti-inflammatory) and mupirocin (anti-bacterial).

As expected (Kong et al., 2012; Chng et al., 2016; Gonzalez et al., 2016), distinct cutaneous microbiota alterations were observed on the lesional skin of AD patients compared with the HC group, with the former showing reduced relative abundances of *Corynebacterium* and *Streptococcus* species. Among the most abundant genera on healthy human skin (Totte et al., 2019), and able to activate particular γδ T cells subsets and repress inflammation in AD patients (Cullen et al., 2015), *Corynebacterium* abundance was inversely related to *S. aureus* in the nares (Totte et al., 2019). The lower abundance of *Streptococcus* species is consistent with previous studies in young children with AD (Kong et al., 2012). Surprisingly, *Propionibacterium* was not one of the representative cutaneous bacteria in the HC group as previous indicated (Gonzalez et al., 2016). We consumed that the phenomena was probably caused by the sequencing bias on different 16S rRNA regions (Guo et al., 2013), and varied life styles in Chinese population, such as different environment and skincare habit (Hulshof et al., 2017).

Also as expected (Kong et al., 2012; Gonzalez et al., 2016), the AD children we studied had substantially reduced cutaneous microbial diversity, similar to adult AD patients (Chng et al., 2016), reflecting the dominance of a small number of taxa (Byrd et al., 2017). However, we did not detect significantly increased *S. aureus* in all AD patients as was previously reported (Kong et al., 2012; Gonzalez et al., 2016; Totte et al., 2019), suggesting that other opportunistic pathogens can dominate the dysbiotic niche. As such, although *S. aureus* is defined as a marker for AD clinical diagnosis (Meylan et al., 2017), our results indicate that the skin microbiota in some AD patients may be dominated by other typically non-resident organisms, including *Pseudomonas* or *Prevotella*, instead of *S. aureus*. These two bacteria commonly colonize AD skin lesions (Kim et al., 2017), and could aggravate inflammatory reactions and cutaneous injury similar to *S. aureus* (Brook, 2002). By stimulating the generation of IL-22, *Pseudomonas* could aggravate inflammation (Kamada et al., 2013), and *Prevotella* are normal cutaneous bacteria in vaginally delivered children (Dominguez-Bello et al., 2010). We speculate that *S. aureus* is only one of several opportunistic pathogens to colonize AD patients, consistent with the 38–74% rate of *S. aureus*-positivity in culture-based studies (Breuer et al., 2002; Park et al., 2013). Moreover, the predominance of different opportunists in AD patients suggests their competition for the same disordered microenvironment (Odell and Flavell, 2016). Since the cutaneous immune system is more tolerant of *S. aureus* than *Pseudomonas* or *Prevotella* (Odell and Flavell, 2016), *S. aureus* may more readily colonize with secondary inflammatory consequences (Byrd et al., 2017), consistent with an earlier report that *S. aureus* density was significantly higher at lesional sites than non-lesional sites in AD patients (Gonzalez et al., 2016).

All AD patients in the present study were prescribed mupirocin to reduce the burden of infection, and cutaneous microbiota alterations were detected in 40 AD.S or AD.ND patients. As expected (Gonzalez et al., 2016), cutaneous microbial diversity normalized and cutaneous injury was reduced in AD.S patients, after the *S. aureus* reduction. These findings were consistent with a randomized study indicating the efficiency of oral antibiotics for skin infections in pediatric AD patients (Huang et al., 2009). In contrast, that decreased cutaneous microbial diversity occurred while the cutaneous lesions recovered, suggests a different mechanism. Since *Pseudomonas fluorescens* is the source of mupirocin (Matthijs et al., 2014) to which other *Pseudomonas* species are resistant (Lynch et al., 2017), mupirocin might relatively or absolutely select for these pathogens. Consistent with the hypothesis of antibiotic selection, increased *Pseudomonas* species were discovered in most of the AD patients after topical mupirocin, paralleling increased *Pseudomonas* representation in severe acne patients after oral treatment with minocycline (Chien et al., 2019). As such, the application of antibiotics in these patients might worsen their cutaneous microbial dysbiosis, and in very young patients might affect normal immunologic maturation (Blaser, 2017). Based on the dichotomous responses to mupirocin, these data suggest that alternative treatments should be considered for AD patients with differing cutaneous microbiota components.

A limitation of the current research is that long-term follow-up of clinical status and microbiome in these AD patients was not obtained. With follow-up investigations, the relationships between cutaneous microbiota compositions and AD relapses can be ascertained, including better understanding of the impact of antibiotics on childhood microbiome development. An important unresolved question is the extent to which the topical application of antibiotics affects the development of microbiome composition and structure at other anatomical sites. To improve understanding pathogenetic mechanisms in AD, we plan to address the following issues in future studies: (i) compare cutaneous metabolites between HC and AD patients (stratifying for AD.S and AD.ND) to learn the associations between specific cutaneous microbiota and metabolites; (ii) to compare skin microbiota changes among AD patients receiving different therapies; and (iii) to assess the effects of the cutaneous treatment on microbiome composition at non-cutaneous sites. Such studies should provide a theoretical basis for assessing approaches to the skin microbiota in AD therapy.

In summary, the study described the skin microbial characteristics for AD children, evaluated the efficacy of mometasone and mupirocin on AD treatment, and showed the differentiating responses of the skin microbiota in AD children. These findings revealed two varied patterns of skin microbial dysbiosis in AD children and provide new tools for AD evaluation and treatment.

## Data Availability Statement

The datasets presented in this study can be found in online repositories. The names of the repository/repositories and accession number(s) can be found in the article/Supplementary Material.

## Ethics Statement

The studies involving human participants were reviewed and approved by the Ethics Committee of Beijing Children's Hospital, Capital Medical University under registration number IEC-C-008-A08-V.05.1. The patients/participants/parents of all children provided their written informed consent to participate in this study.

## Author Contributions

YLiu, SW, and WD wrote primary and revised versions of manuscript and main data analysis. YLia, CS, YuL, LJ, and YB collected sample. ZG, YiL, and DL shared in analyzing the data. SL provided data analysis consultation. MB and Y-WT provided critical review and edits of manuscript drafts. Y-WT and LM conceived project idea, collaborations, and design. All authors read and approved the final manuscript.

## Conflict of Interest

The authors declare that the research was conducted in the absence of any commercial or financial relationships that could be construed as a potential conflict of interest.

## Supplementary Material

Supplementary File 1 SCORAD score for enrolled subjects.

Supplementary File 2 Distribution of cutaneous microbiota in all subjects at the genus level.

Supplementary File 3 Group information for participants after hierarchical clustering.
